# Comparison of Nocturia Response to Desmopressin Treatment between Patients with Normal and High Nocturnal Bladder Capacity Index

**DOI:** 10.1155/2013/878564

**Published:** 2013-10-07

**Authors:** Tine Hajdinjak, Jurij Leskovar

**Affiliations:** ^1^Division of Urology, Department of Surgery, General Hospital Murska Sobota, 9000 Murska Sobota, Slovenia; ^2^Department of Urology, UKC Maribor, 2000 Maribor, Slovenia

## Abstract

*Objective*. To compare efficacy of desmopressin for treatment of nocturia between patients with normal and high nocturnal bladder capacity index (NBCi). *Methods*. Retrospective analysis of adult patients treated with desmopressin for nocturia. Patients were analyzed according to high or normal NBCi value before treatment. *Results*. 55 patients were identified, aged 49–84, 47 males, 8 females, who started desmopressin 0.2 mg nocte between 2009 and 2011. Two groups (N: normal and H: high NBCi) were similar regarding number, gender, age, 24 h urine volume, and nocturnal urine volume. On treatment, nocturnal volume decreased by mean of 364 mL. Number of nightly voids decreased in N group from 3.11 to 1.50, in H from 3.96 to 1.44. Nocturnal polyuria and nocturia indices also decreased significantly. NBCi remained the same in N group (0.56 on therapy) and in H group decreased to mean 0.63. All on-treatment values were statistically similar in N and H groups. Pretreatment differences were abolished with treatment. NBCi was significantly correlated to nocturia reduction—larger reduction was observed in patients with higher NBCi. In 8/55 patients, hyponatremia was detected, but without clinical consequences. *Conclusions*. The results indicate that the effectiveness of desmopressin on nocturia is not dependent upon the patient's pretreatment NBCi.

## 1. Introduction

Desmopressin is used universally for the treatment of pediatric nocturnal enuresis and for diabetes insipidus. Its role in the treatment of nocturia was initially reserved for highly selected cases and neurogenic patients [1]. Desmopressin's wider use as a primary drug for managing nocturia evolved only recently, as nocturia became a significant disease entity with its own chapter in Campbell's Urology and is now recognized as one of most bothersome lower urinary tract symptoms (LUTSs) [2]. Use of desmopressin in patients with nonneurogenic LUTS was included in European Association of Urology (EAU) guidelines in 2010 and is still not an approved indication in the USA. Recommendations claim it “can be used” for nocturia “based on a polyuric background,” as its mechanism of action is to reduce nocturnal polyuria. Whether desmopressin is equally effective in patients with reduced bladder capacities, both with and especially without nocturnal polyuria have been unclear, and hence we investigated whether the response to desmopressin differs between patients with decreased and normal nocturnal bladder capacities.

## 2. Materials and Methods

Consecutive patients who were prescribed desmopressin for nocturia between 2009 and 2011 by a single urologist (Jurij Leskovar) were retrospectively identified and their charts were reviewed. The principles outlined in the Declaration of Helsinki were followed. All of the patients who were prescribed desmopressin for nocturia were adults and were able to comply with the treatment requirements. The criteria for desmopressin prescription were similar to those given in published trials [3]. Specifically, all patients were questioned about nocturia; those who reported having to void at least twice nightly and that it bothered them, affected their daily life, and interfered with their sleep were evaluated further. Their medical history was checked, focusing on identification of sleep and urinary problems, fluid intake, cardiac problems, medications, and other conditions that might account for excessive nocturia and potential contraindications for desmopressin prescription. Urinalysis and urine culture were performed, and postvoid residual urine and laboratory values (i.e., serum urea nitrogen, creatinine, sodium, and potassium levels) were recorded.

Patients identified with no treatable conditions or contraindications for desmopressin prescription (e.g., confused state, urinary tract infection, shift work, clinically relevant cardiac failure, hyponatremia, uncontrolled hypertension, or significant renal disease) were asked to complete a 48-h frequency volume chart (FVC), including the time and volume of each void, bedtime, and time of rising. Patients with FVC-confirmed nocturia at least twice nightly were prescribed desmopressin according to the European (Slovenian) product registration, which allows prescription not only for nocturia treatment in cases of nocturnal polyuria, described as urine production greater than one-third of the patient's 24-h urine production, but also for nocturia, described as urine production greater than the nightly bladder capacity. There was no upper age limit. While on therapy, patients were instructed to drink only enough to satisfy their thirst from 1 h before until 8 h after taking their dose of desmopressin. When patients were on loop diuretics, they were advised to take them 6–8 hours before bedtime. Those already taking medications for benign prostatic hyperplasia (alpha-blockers) or urinary frequency (anticholinergics) were able to get desmopressin if their condition was stable, no further improvement from those drugs was expected, and they had experienced at least two nocturia episodes at screening while using the prescribed drugs. The patients were required to have their serum sodium checked at weeks 1 and 3 and were reevaluated at 2 months after beginning desmopressin therapy. In the week prior to control evaluation, the patients were requested to complete another 2-day FVC.

In total, 55 eligible patients were identified (age 49–84 years, median 69 years; 47 males, 8 females) who had been prescribed 0.2-mg desmopressin in tablet form (Minirin, Ferring) to be taken at bedtime. Of these, eight patients were taking anticholinergic medications, seven were on alpha-blockers, and six were on 5-alpha reductase inhibitors.

The FVC provided details regarding the actual number of nightly voids (ANV), nocturnal urine volume (NUV), and maximum voided volume (MVV; i.e., bladder capacity). These data were used to calculate the nocturia index (Ni: NUV/MVV; normal, <1.5) [4], nocturnal polyuria index (Npi, NUV/24-h volume, >35% indicates nocturnal polyuria), and nocturnal bladder capacity index (NBCi). An unrounded NBCi calculation {ANV − [(NUV/MVV) − 1]} was used, with a cut-off value of 1.3, which was consistent with a recent study suggesting that rounding of NBCi values is not appropriate and that 1.3 is an appropriate cut-off; higher values indicate reduced bladder capacity as a cause of nocturia [5]. Other nocturia-related bladder indices were calculated according to International Continence Society definitions [6, 7]. The responses to desmopressin treatment were compared between patients whose pretreatment urinary NBCi was high (i.e., >1.3; group H) and normal (i.e., ≤1.3; group N). Individual end points, which were derived from FVCs, nocturia indices, quality of life score, and side effects (i.e., changes in serum sodium), were compared between the two groups. A clinical response was defined as a >50% reduction in the number of nocturnal voids (ANV) and improvement in the period of undisturbed sleep. The factors predicting ANV improvement were also evaluated using pretreatment FVC data.

Safety was assessed by evaluating the incidence of adverse events and changes in biochemical values. Patients were specifically questioned to elicit possible symptoms (headache, nausea, vomiting, fatigue, dizziness, ataxia, or weight gain). 

Statistical analyses were performed using R version 2.13 (R Foundation for Statistical Computing, Vienna, Austria).

## 3. Results

Three patients stopped the treatment within 2 months due to a lack of efficacy (one with a normal NBCi and two with a high NBCi; one female, two males). The data of the remaining 52 patients were divided into two groups according to their pretreatment NBCi (i.e., N and H). The number of patients and their gender, age, 24-h urine volume, NUV, and Npi did not differ between the two groups. The nocturia index was higher and ANV was lower in group N than in group H. The pretreatment data are presented in Table 1.

**Figure 1:**
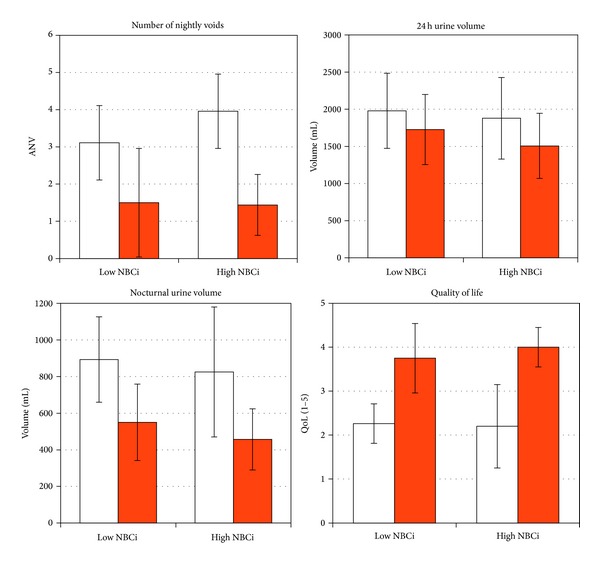
Effect of desmopressin therapy according to normal or high pretreatment nocturnal bladder capacity index (NBCi) on number of nightly voids, 24-h urine volume, nocturnal urine volume, and quality of life. Each pair: first column—before therapy, second column—on therapy. All pre- versus on-therapy values were statistically significantly different (also 24 h urine volume, where p was 0.03 for normal NBCi and 0.001 for high NBCi). On therapy, comparison of each two groups showed no significant differences for all four measures.

**Figure 2:**
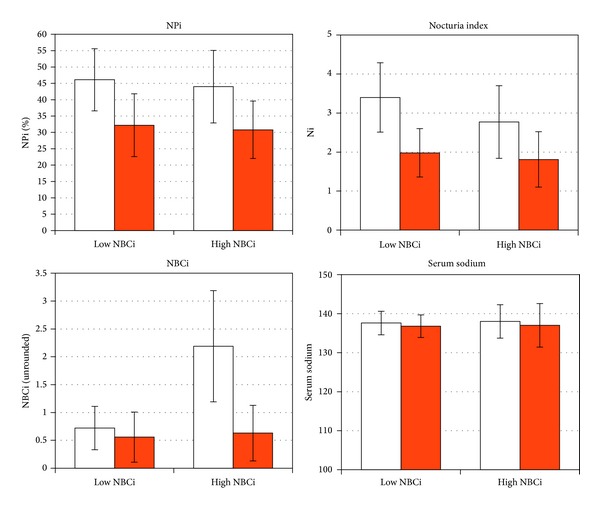
Effect of desmopressin therapy according to normal or high pre-treatment nocturnal bladder capacity index (NBCi) on nocturnal polyuria index (NPi), nocturia index (Ni), NBCi, and serum sodium. Each pair: first column—before therapy, second column—on therapy. Pre- versus on-therapy values for NPi and Ni were statistically significantly different with *P* < 0.0001. NBCi in normal pre-treatment NBCi group remained the same on treatment (*P* = 0.28) and decreased significantly in high pre-treatment group (*P* < 0.0001). Regarding serum sodium, there were no difference between pre-treatment and on-treatment values, for high NBCi group *P* = 0.93, for normal NBCi group *P* = 0.066. On therapy, comparison of each two groups showed no significant differences for all four measures.

During the treatment period, mean NUV decreased from 893 to 550 mL in group N and from 825 to 457 mL in group H. ANV decreased from 3.11 to 1.50 in group N and from 3.96 to 1.44 in group H. Npi decreased from 46% to 32% in group N and from 44% to 31% in group H. Ni decreased from 3.4 to 1.98 in group N and from 2.77 to 1.81 in group H. The quality of life (assessed on a scale of 1–5) increased from 2.26 to 3.75 in group N and from 2.2 to 4.0 in group H. Differences between pretreatment and on-treatment values were highly statistically significant. While mean NBCi remained the same in group N (0.56 on therapy), it decreased in group H to 0.63. None of the on-treatment values differed significantly between the two groups. Pretreatment differences (in ANV, Ni, and NBCi) were abolished with treatment. The observed and calculated parameters in both patient groups before and after treatment and the associated statistics are presented in Figures 1 and 2.

Mean nocturnal bladder capacity (maximal voided volume at night) did not change with treatment in either group N (pretreatment = 259 mL, on-treatment = 262 mL) or group H (264 and 242 mL, resp.). 

Average nocturnal micturition volume, calculated as NUV divided by number of nightly voids plus 1 (morning void), increased significantly in group H with treatment (from 172 to 211 mL, paired *t* test, *P* = 0.024; mean increase in volume = 40 mL, 95% confidence interval = 6–74 mL) and remained unchanged in group N (221 and 222 mL before and on-treatment, resp.).

Clinical response (ANV reduction by at least 50%) was observed in 73% and 62% of patients in groups H and N, respectively (*P* > 0.1). Overall, nocturia was normalized in 51% of the patients (to one or zero episodes per night). Npi was normal (below 35%) in only 12% of the patients before treatment, but in 62% of the patients when on treatment. The duration of undisturbed sleep increased from 107 ± 33 min (mean ± SD) before treatment to 250 ± 91 min when on treatment. None of the patients were able to achieve 5 h of undisturbed sleep before treatment, but 39% of patients achieved this goal when on treatment. It was found that the pretreatment ANV was the best predictor of a treatment-induced decrease in ANV with multivariate regression, compared to any combination of bladder indices (*R* ^2^ = 0.65). A higher pretreatment ANV indicates a greater expected improvement with treatment.

The following side effects of the treatment were reported by 8 of the 55 patients (14.5%) and were described as mild to moderate in severity: abdominal cramps (*n* = 2), peripheral edema (*n* = 1), headache (*n* = 2), dizziness (*n* = 1), and voiding problems (*n* = 2). Hyponatremia was identified in 8 of the 55 patients (14.5%). In seven patients (all older than 65 years), control sodium values were between 132 and 134 mmol/L (reference values 135–145 mmol/L). There was one case of significant hyponatremia (i.e., <125 mmol/L) in a 76-year-old male who claimed to have no symptoms and insisted on being allowed to continue the treatment. After normalization of serum sodium values, the dose in this patient was reduced from 0.2 to 0.05 mg (at the time no MELT formulation was available in our country). 

## 4. Discussion

Nocturia at least twice nightly is reported by 35% of the population over 60 years of age [8] and by 69% of males and 49% of females over 80 years of age [9]. This is accompanied not only by a reduction in quality of life [10, 11] but also by large economical losses [12], increased morbidity [13], and even reduced survival [14], although probably as a significant predictor rather than as a primary cause [15]. Nocturia and its potential treatments therefore warrant investigation [16].

Desmopressin is the first-choice treatment for bothersome nocturia [17] in cases without global polyuria (i.e., <40 mL/kg), with nocturnal polyuria (Npi > 35%), where behavioral modifications and other specific treatments fail [18, 19]. Desmopressin might not be effective in patients with a reduced global or nocturnal bladder capacity. A single-arm study involving a Korean cohort showed that desmopressin is generally effective in patients with mixed nocturia, where NBCi was >1 [20]. The present study supplemented this by comparing responses to desmopressin between patients with and without a decreased nocturnal bladder capacity, implementing a new definition for normal NBCi [5] and a longer observation time than in previous studies, and focusing on a Caucasian population.

The main effect of desmopressin is a reduced NUV; we observed an average reduction of 41%. MVVs (bladder capacities) were not improved. Average voided volume at night increased in group H and remained the same in group N. The small but statistically significant 40-mL increase represents a 20% improvement that also caused average voided volume at night to become similar in the two groups. Average nighttime voided volumes still remained lower than both the bladder capacities (MVVs) and the maximal observed nocturnal bladder volumes. Observed desmopressin-induced 20% increase in average nighttime voided volume in patients with increased NBCi before treatment may have been due to a slower rate of bladder filling, as was found in a study involving pigs [21]. In addition, desmopressin modulates the activity of the brainstem micturition center, as discussed by Lee et al. [20] in studies on rats [22]. This underscores the multifactorial causative role of bothersome nocturia, and suggests the need for combined treatments. For example, in addition to combinations that address the different mechanisms of nocturnal polyuria [23], future studies should focus on the use of anticholinergic medication or other measures to increase bladder capacity (e.g., low-dose botulinum toxin, beta-3 agonists, or imipramine) in combination with desmopressin. Our study included insufficient patients with anticholinergic medications to allow conclusions to be drawn regarding the place and role of combinations of desmopressin and anticholinergics.

We found that not only nocturnal urine volume, but also 24-h urine volume decreased significantly (by an average of 16%) in both groups of patients treated with desmopressin. It was claimed before, also in EAU guidelines, that desmopressin therapy does not reduce 24-h urine volume [24, 25]. While the observed reduction might have been influenced by behavioral factors, such measures (e.g., drinking only to satisfy thirst) are important for safety reasons with desmopressin therapy itself and should be regarded as an integral part of desmopressin therapy for nocturia [25]. It is possible that our inclusion of more subjects (*n* = 55) than in the reference study (*n* = 17) [25] contributed to our ability to identify a moderate (16%) but significant decrease also in 24-h urine volume. Furthermore, in older patients, delayed compensatory urine output preceding the next dose of desmopressin may have played a role [26].

The main limitations of our study are its retrospective design and lack of a placebo group. The lack of a placebo group means that our findings do not directly support the use of desmopressin as a therapy for nocturia; however, the data do suggest that the antidiuretic can be used in patients with nocturnal polyuria and those with diminished nocturnal bladder capacity. Another limitation is the small number of female subjects, which reflects the patient population in our practice. There is evidence that desmopressin therapy is at least equally effective for females than for males: 88% of females and 67% of males reported a reduction of more than 50% in the number of nightly voids (*P* = 0.18). Apparently better results in our few female patients compared to the males (although not statistically significant) may be explained by the specific dosing protocol that was used (a 0.2-mg fixed dose) and the known better sensibility to desmopressin among females [27]. 

We did not use dose titration, but fixed low dose protocol. One possible advantage of a relatively low dose is improved safety, since higher doses are associated with a higher incidence of hyponatremia [28]. A few marginal cases and one significant case of hyponatremia were observed even with this low dose. Prescribing even 0.2 mg of oral desmopressin requires strict serum sodium control prior to and during treatment [29]. Patients should receive clear instructions regarding the need for reduced fluid intake to monitor their weight and to cease desmopressin medication if they develop any acute illness, until their situation stabilizes and their doctor checks their sodium level. Intermittent dosage (for example 3 times weekly) or better reduced dose (half or even one-quarter of a 0.2-mg tablet) may also be useful in patients with a good response and no side effects, in whom there is a decrease in serum sodium. A MELT formulation may further improve safety by reducing the overall drug dose. A lower dose may be necessary for female patients [27].

Among the patients identified with bothersome nocturia who were found to be suitable for desmopressin treatment and who were actually prescribed desmopressin, 95% responded to the treatment and 51% of those exhibited normalization of their nocturia (i.e., achieved zero or one urination per night), irrespective of whether the main direct cause of their nocturia was nocturnal polyuria or a decreased nocturnal bladder capacity.

## 5. Conclusions

Desmopressin is beneficial in patients with an increased NBCi at least as much as in patients with normal NBCi values. Patients with a higher pretreatment ANV or higher NBCi values can expect desmopressin to induce a greater absolute decrease in that parameter.

## Figures and Tables

**Table 1:** Characteristics of patients who were prescribed desmopressin. Data were taken from medical history and frequency-volume chart they filled before treatment start. They are divided into two groups according to nocturnal bladder capacity index (NBCi): increased NBCi (above 1.3) and normal NBCi (1.3 or less).

	NBCi 1.3 or less	NBCi above 1.3	*P*
N	26	26	1
Age—years (med, range)	70.9, 48–82	67.9, 48–84	0.23
Female (*N*, %)	2, 8%	5, 19%	0.22 (chi-sq)
24 h Vol—mL (med)	1935	1780	0.50
NUV—mL (med)	855	800	0.42
ANV—*N* (med, range)	3, 2–6	3, 2–8	0.014
NPi (med, range)	46, 30–76	42, 22–67	0.47
Ni (med, range)	3.3, 2.3–6.5	2.5, 1.5–4.9	0.018
NBCi (med, range)	0.67, 0–1.3	2.04, 1.33–6.50	<0.0001
